# Informing the Development of a Digital Health Platform Through Universal Points of Care: Qualitative Survey Study

**DOI:** 10.2196/22756

**Published:** 2020-11-26

**Authors:** Michael P Craven, Jacob A Andrews, Alexandra R Lang, Sara K Simblett, Stuart Bruce, Sarah Thorpe, Til Wykes, Richard Morriss, Chris Hollis, The RADAR-CNS Consortium

**Affiliations:** 1 NIHR Mindtech Medtech Co-operative Institute of Mental Health University of Nottingham Nottingham United Kingdom; 2 Bioengineering Research Group Faculty of Engineering University of Nottingham Nottingham United Kingdom; 3 NIHR Nottingham Biomedical Research Centre University of Nottingham Nottingham United Kingdom; 4 Division of Psychiatry and Applied Psychology Institute of Mental Health, School of Medicine University of Nottingham Nottingham United Kingdom; 5 Human Factors Research Group Faculty of Engineering University of Nottingham Nottingham United Kingdom; 6 Institute of Psychology, Psychiatry and Neuroscience King's College London London United Kingdom; 7 Patient Advisory Board RADAR-CNS London United Kingdom; 8 NIHR Biomedical Research Centre for Mental Health South London and Maudsley NHS Foundation Trust London United Kingdom; 9 London United Kingdom

**Keywords:** epilepsy, multiple sclerosis, depression, wearable electronic devices, remote sensing technology, health personnel, mobile phones, mHealth, eHealth

## Abstract

**Background:**

Epilepsy, multiple sclerosis (MS), and depression are chronic conditions where technology holds potential in clinical monitoring and self-management. Over 5 years, the Remote Assessment of Disease and Relapse - Central Nervous System (RADAR-CNS) consortium has explored the application of remote measurement technology (RMT) to the management and self-management of patients in these clinical areas. The consortium is large and includes clinical and nonclinical researchers as well as a patient advisory board.

**Objective:**

This formative development study aimed to understand how consortium members viewed the potential of RMT in epilepsy, MS, and depression.

**Methods:**

In this qualitative survey study, we developed a methodological tool, universal points of care (UPOC), to gather views on the potential use, acceptance, and value of a novel RMT platform across 3 chronic conditions (MS, epilepsy, and depression). UPOC builds upon use case scenario methodology, using expert elicitation and analysis of care pathways to develop scenarios applicable across multiple conditions. After developing scenarios, we elicited views on the potential of RMT in these different scenarios through a survey administered to 28 subject matter experts, consisting of 16 health care practitioners; 5 health care services researchers; and 7 people with lived experience of MS, epilepsy, or depression. Survey results were analyzed thematically and using an existing framework of factors describing links between design and context.

**Results:**

The survey elicited potential beneficial applications of the RADAR-CNS RMT system as well as patient, clinical, and nonclinical requirements of RMT across the 3 conditions of interest. Potential applications included recognition of early warning signs of relapse from subclinical signals for MS, seizure precipitant signals for epilepsy, and behavior change in depression. RMT was also thought to have the potential to overcome the problem of underreporting, which is especially problematic in epilepsy, and to allow the capture of secondary symptoms that are not generally collected in MS, such as mood.

**Conclusions:**

Respondents suggested novel and unanticipated uses of RMT, including the use of RMT to detect emerging side effects of treatment, enable behavior change for sleep regulation and activity, and offer a way to include family and other carers in a care network, which could assist with goal setting. These suggestions, together with others from this and related work, will inform the development of the system for its eventual application in research and clinical practice. The UPOC methodology was effective in directing respondents to consider the value of health care technologies in condition-specific experiences of everyday life and working practice.

## Introduction

### Background

Prior work has recognized the need for health information technologies (HITs) to consider socio-technical facets used over time and the varying trajectories experienced within and between health care services [1]. Previous work in mobile health has indicated how technologies may be able to alter the relationship and dynamics of clinician-patient interactions, where previously the clinician has been seen as the main decision maker and expert provider of information and knowledge [2,3]. The introduction of monitoring, measurement, and communication technologies should, in principle, support informed patient-clinician partnerships and greater shared decision making, although there may be barriers to their use [4,5].

The importance of ensuring that the requirements of all users are considered early in the design process of products and services is well established. The benefits of user-centered design approaches underpin improved usability and adoption, and human factors and ergonomics is a key discipline in this area [6]. In particular, through a systems approach to design, the added value of novel technologies can be understood when these advances enable new ways of working, speed up or make existing practices easier, or enhance the user experience [7]. In addition, this perspective supports the inclusion of all users within a system and aims to understand the multiple socio-technical interactions of a new intervention, whether they are human-to-human interactions, human-to-technology interactions, or any permutation. Despite this knowledge and a regulatory landscape supporting the inclusion of all stakeholders during design and development, there is evidence that HITs producers are still responding to technology push, and the requirements of all users are not necessarily prioritized during system development [8].

In this study, we aim to investigate how the introduction of a remote measurement technology (RMT) platform might alter current practices in care pathways for people with epilepsy, multiple sclerosis (MS), or depression and identify where, how, and why clinicians and patients may derive value from the RMT platform once in practice.

### Study Technology Overview

The Remote Assessment of Disease and Relapse - Central Nervous System (RADAR-CNS) RMT platform under consideration in this study is being designed for use in the clinical management and patient self-management of 3 long-term conditions: epilepsy, MS, and depression. However, the long-term goal is for a flexible, ubiquitous platform that can be adapted to support the management of a wide range of conditions spanning physical and mental health domains as well as use in research.

The RMT system in development consists of wearables and mobile phone apps run on consumer mobile devices. A passive remote measurement technology software component processes sensor data such as activity and location. Passive data collection systems have been used in various applications in general health [9] and mental health in particular [10]. An active remote measurement technology component processes user-supplied data entries. This includes data from short questionnaires completed at regular intervals on mobile devices after notification to the user, usually termed ecological momentary assessment or experience sampling method, and other prompted input such as voice samples. Longitudinal data sets are collected from individual users with the aim of processing through predictive algorithms to understand whether early detection and warning of relapse and/or disease change (worsening or improvement) can be identified via RMT.

**Figure 1:**
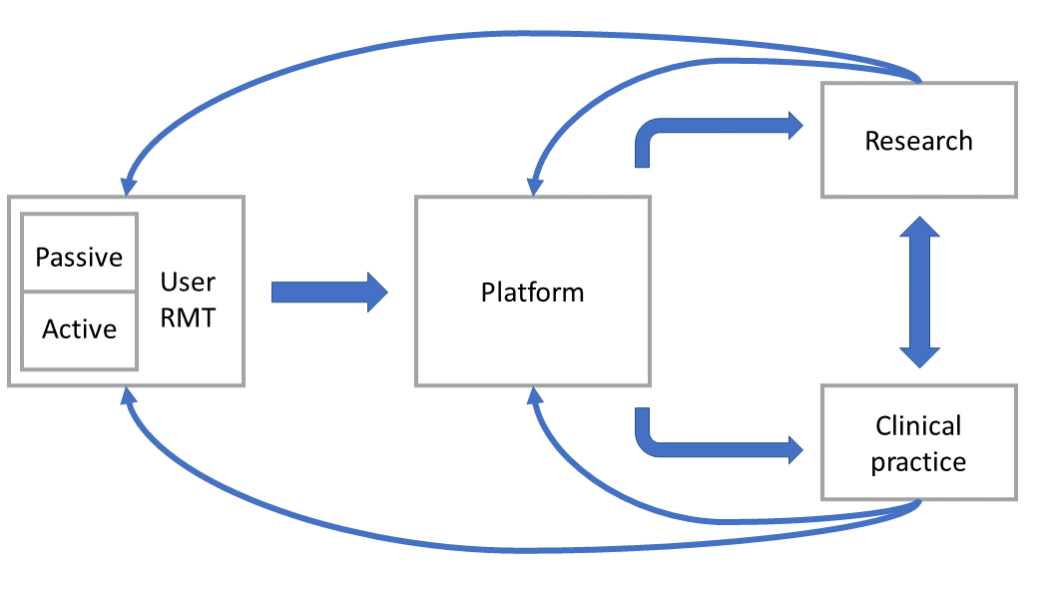
Diagram showing how the platform under development is intended for use in clinical practice and research. The platform is supplied with data from passive and active remote measurement technology worn or carried by patient users, as instructed by the clinic or in research protocols. Use in research and practice will inform further development of the platform. RMT: remote measurement technology.

Furthermore, a number of translational work packages exist to probe the user acceptability and fit of RMT to health care systems and in the everyday lives of health care professionals and patients. All work is at a formative stage, and no system has been introduced into health care practice at this stage. In the initial iteration of the system, which is used in observational research studies, there is no predictive element and no feedback from the system to the patient, although these are features that have the potential to be developed and included in the longer term. Figure 1 illustrates how use in research and practice will inform further development of the platform.

### Scenario-Based Design

Use cases and design scenarios have been used in design processes since the early 1990s [11] and constitute a key tool when exploring technology users’ needs and the nature and requirements of cooperative work systems [12,13]. Within the field of health care, use case scenarios have been applied in investigations into clinical decision making and associated tools and aids [14-16] and in further understanding knowledge sharing in distributed working practices [17]. Scenarios help participants to envisage how new technologies might be implemented in practice where these are not yet common. Scenarios are useful because they encourage consideration of multiple facets of a situation and foster elicitation of views that can inform the development of tools and services. However, use case scenarios tend to work best in task-specific contexts, where parameters are clearly defined and the outcome of use is predictable [18].

Conversely, digital health care systems tend to be complex in nature, with multiple users, addressing multiple medical conditions, and with unpredictability in use [19]. In this paper, we describe the use of a new methodology, based on use case scenarios, to inform the design of the RADAR-CNS RMT system, which is to be used in research and ultimately in clinical practice. The system is required to work across multiple conditions, collecting data from patients who have differing requirements from health care professionals treating them. Thus, we required a methodology to inform the design of the system to ensure applicability within and between conditions. The methodology was also required to allow participants in the research program (clinicians and patients) to contribute from their own expertise and experience, without having to adapt the research method for each condition. We call our approach universal points of care (UPOC).

## Methods

### Approach

A survey was developed to elicit opinions from European health care professionals and researchers within the consortium and the patient advisory board (PAB), which was made up of individuals with lived experience of the 3 conditions of interest. The aim was to focus on the potential and practicalities of RMT use in everyday practice. In particular, we sought to carry out a preliminary investigation into the current practices across the 3 central nervous system (CNS) conditions. The intention was to provide space for clinical opinion to explore prospectively how practice would (or could) be affected by the introduction of the RADAR-CNS RMT system and to uncover needs and concerns about this. The approach taken was to ask participants about a series of preidentified points of care common across the 3 conditions, and then to elicit further points of care that may be affected by the introduction of the RMT system. Generic clinical scenarios were identified through expert elicitation and exploration of clinical pathways. The developed scenarios became UPOC exemplars, providing the basis for an inquiry that would be relevant to multiple clinical conditions.

### Development of UPOC Exemplars

Clinical pathways provide a framework through which changes relevant to the implementation of new technologies and devices can be considered. Clinical pathways represent care management within a clinic setting [20] and can also refer to the planned, managed delivery of care over time and by care teams in a range of clinical settings, as per the definition of clinical pathways provided by the European Pathway Association [21].

The UPOC were informed by consultation of academic and gray literature on clinical pathways in each of the following conditions: a review of treatment pathways for MS [22], an international audit of epilepsy services worldwide [23], and the European Psychiatric Association’s guidance on psychotherapy in chronic depression [24], among other work. The UPOC were derived by reviewing existing clinical pathways and decision points of the 3 target clinical areas via examination of the National Institute for Health and Care Excellence care pathway maps [25]. These maps consist of interactive flowcharts presenting details of the recommendations for managing patients of different groups (eg, by age), including the recommended steps involved in diagnosis and treatment. Here, we focused on the pathways for epilepsy, MS, and depression. Knowledge was also gained from informal discussions with clinicians in the consortium, focusing on personal experiences of care provision and reflecting on the current clinical pathways for epilepsy, MS, and depression.

Once identified, the UPOC were designed into the survey, and participants were asked to consider how those UPOC might be experienced both without and with an RMT system in place, to contrast current care provision with future care provision when the RADAR-CNS RMT system is deployed. It was decided that participants would also be invited to describe any additional use scenarios that they believed would be encountered in care delivery or receipt with the use of RMT.

### Participants

A convenience sampling method was used to recruit members of the consortium to take part in the survey, some at an annual meeting that took place in April 2018 and some shortly after. To encourage engagement, a panel session was held at the annual meeting, with presentations by 3 clinicians and 3 members of the PAB discussing value propositions of novel health care technologies. This also included details on the content, aims, and importance of the survey. Delegates of the meeting were asked to complete the survey immediately afterward. Members of the consortium were freely able to take part (or not). The survey was administered via a web-based interface for ease of access and to provide the opportunity for remote completion. PAB participants were given the option to complete the survey by hand, by email, or by phone—in the latter case, a researcher made hand-written notes on a printed survey form for each survey administered. Due to their involvement in the research program, all the participants had baseline knowledge of the RADAR-CNS RMT system and its potential functional capabilities, for example, remote monitoring of disease, distributed and computer-supported delivery, and receipt of care and personalized medicine. Participants were asked to respond in relation to the condition on which they had most knowledge.

### Survey Sections

The survey was divided into 6 sections, A-F, as follows. All respondents were encouraged to answer all questions. The survey instrument is available in Multimedia Appendix 1. An overview of survey sections is provided in Table 1. These are shortened titles of the survey sections; more comprehensive titles were used in the survey itself, and they can be found in Multimedia Appendix 1.

**Table 1:** Survey sections.

Sections	Survey sections
Section A	Respondent information
Section B	Promise of RMT^a^
Section C	UPOC^b^ 1-6 comparing existing and future potential care
Section D	Respondent-provided UPOC
Section E	Use of RMT data
Section F	Reflections and comments

^a^RMT: remote measurement technology.

^b^UPOC: universal points of care.

Section A of the survey collected demographic information about the respondents, including names; geographical location; role in the partnership; and where relevant, place of work, clinical or research specialty, and for the PAB respondents, their clinical condition.

Section B was designed to gather general opinion about the promise of RMT.

Section C presented the UPOC to participants for them to consider how these occurred in their experience *before RADAR-CNS* and how they may occur *after RADAR-CNS*. Questions in the survey text prompted consideration of various aspects of the UPOC: *Where does the interaction take place? Are any communications in real time or is there a delay? What are the positives about this way of interacting? What are the negatives about this way of interacting?* Six UPOC that were identified and used in the survey are presented in Textbox 1.

**The 6 universal points of care for which the respondents were asked to provide details about the scenarios before and after the introduction of remote measurement technology.**

Patient sharing data with clinician

- A patient has been monitoring how they feel (activity, mood, sleep, etc) for 2 weeks. They have some concerns and would like to share their data with their clinician. How might this happen?

Relapse detection (symptomatic)

- A patient is not feeling well and might be on the path to relapse. How do you (patient or clinician) try to understand if a relapse is imminent or occurring?

Relapse detection communication (recently diagnosed patient)

- The patient might be headed for a relapse. The clinician and the patient have not been working together for very long, and the patient is still getting used to their diagnosis. How would this be communicated?

Relapse detection communication (patient diagnosed for a long time)

- The patient might be headed for a relapse. The clinician and the patient know each other well, and the patient has been living with the condition for a long time. How would this be communicated?

Medication selection or dosing (recently diagnosed patient)

- The patient’s medication needs modification. What is the best way of informing them of this decision and providing them with information?

Medication selection/dosing (patient diagnosed for a long time)

- The patient’s medication needs modification. What is the best way of informing them of this decision and providing them with information?

In section D, respondents were asked to offer up to 2 additional UPOC of their own, inviting them to consider “other situations where use of the technology might change your decisions, behaviours or experiences or the way in which you interact with other people (clinicians, patients, other healthcare professionals).” In section E, respondents were asked to consider the possibility of 2 more speculative UPOC specific to RMT implementation: (1) stratification of patient risk for relapse and (2) dealing with different patient responses to treatment. During analysis, these were then rated as either *considered useful*, *considered possible*, *would need more research*, or *have concerns*. In section F, any final additional comments were collected.

### Data Analysis

Data were gathered via a web-based interface, Bristol Online Surveys [26], which collected and grouped the raw data of respondents for section A-F of the survey. The raw data consisted of single sentences or short paragraphs written by individual respondents to each question that was posed. Thematic analysis [27] was carried out on the collected data. Responses were coded by 2 coders in 2 iterations, whereby each individual response (or excerpt from a paragraph) was given a descriptive term to create a set of initial themes for each section of the survey (refer to the *Results* section for examples of these terms). The responses or excerpts coded with the initial themes were cut and pasted into tables that were reviewed and combined into a smaller set of final themes for that section. During the transfer to tables, data from the different clinical specialties were color coded to help identify condition-specific patterns. In the presentation of the final thematic coding structure, this approach enabled analysis within and between conditions. Data from the PAB were themed separately by a third coder in a single iteration after analysis of the results. The coding was based on the themes found in the web-based survey, with the intention to draw out commonalities and differences from the health care professionals.

Responses within the final themes were analyzed further to qualify the benefits participants saw to the introduction of RMT, using the following framework of descriptors adapted from Sharples et al [7]:

- Enabler: The introduction of the device/system permits a new process or function to be possible (ie, it is not currently possible).
- Enhancer: The introduction of the device/system permits an improved outcome (ie, it has an impact on effectiveness).
- Facilitator: The introduction of the device/system makes a process or function easier (ie, it has an impact on satisfaction and adherence).
- Catalyst: The introduction of the device/system speeds up a process or function (ie, it has an impact on efficiency).

## Results

### Overview

The surveys returned were generally well completed. No portions of the survey were consistently ignored, although in response to some questions, participants signposted to earlier responses given, indicating that the answer was applicable to multiple UPOC. Furthermore, not all respondents provided their own suggested use cases in section D.

### Section A: Respondent Information

Table 2 shows number of respondents to the survey from different roles within the consortium. As the health care system was the main focus of the work package, survey responses from translational, technical, and clinical respondents were analyzed separately from responses provided by the 7 members of the PAB, results for which are presented at the end of this section.

**Table 2:** Respondent roles in the consortium.

Role in research program		Participants (n=28), n (%)
**Translational**		
	Health service researchers (clinical pathways, patient and public involvement, regulatory requirements)	3 (11)
**Technical**		
	Health technology researchers (devices and software platform, data analysis, biosignatures)	2 (7)
**Clinical**		
	Epilepsy	4 (14)
	Multiple sclerosis	8 (29)
	Depression	2 (7)
	Clinical harmonization	2 (7)
**Patient advisory board**		
	Epilepsy	2 (7)
	Multiple sclerosis	3 (11)
	Depression	2 (7)

### Section B: Promise of RMT

Table 3 shows a summary of the 9 final themes arising from section B. Further details on the contents of each theme are available in Multimedia Appendix 2. Responses to the section on the promise of RMT (potential uses) were analyzed thematically, where 15 initial descriptive terms were attached to the qualitative data and were merged to form a shorter list of 9 final themes, which are listed in Table 2. The themes were generally aligned to the purposes of the study and revealed aspects that were clinician focused as well as reflecting on patient behaviors and experiences. The themes also revealed opportunities for use of the platform, for example, roles in clinical trial design and clinical decision making as well as raising concerns for their use.

**Table 3:** Themes derived from section B concerning the promise of remote measurement technology.

Final theme	Enabler	Enhancer	Facilitator	Catalyst
Relapse prediction	✓^a^	—^b^	✓	—
Clinical verification	✓	✓	—	—
Monitoring potential	✓	✓	—	✓
Patient behavior	✓	✓	✓	—
User experience	✓	—	—	—
Changes to treatment approach	✓	—	—	—
Clinical decision making	✓	—	—	—
Clinical trials design	✓	—	—	—
Device issues	—	—	—	—

^a^Present in transcript.

^b^Not present in transcript.

We assigned relevant qualifiers to responses under each theme (*enabler*, *enhancer*, *catalyst*, and *facilitator*, as described above). For example, under the theme of *clinical verification*, participants suggested that the RADAR system could *enable* the measurement of deterioration of motor system in progressive MS. Under *monitoring potential*, respondents indicated that the system could *enhance* patient management through faster and more detailed ecological assessment of emotional and physical state. In relation to research, respondents suggested that data collected using the RADAR system could be used to detail the characterization of the progression of MS and that this could *enable* new designs of clinical trials.

The results demonstrate that participants largely viewed RMT as *enabling* new processes or functions that were not currently possible. Fewer responses described ways that RMT would enhance or *facilitate* care provision, and only one provided an example of how RMT could speed up, or *catalyze*, existing processes (Table 3).

One theme, *device issues*, did not fit with the classification of these qualifiers; however, it was considered important to include this in the analysis, as this theme captured some of the practical challenges associated with use of these devices, for example, the accuracy of activity trackers. Device issues have been explored in further detail elsewhere in the consortium [28,29].

### Section C: UPOC 1-6 Comparing Existing and Future Potential Care

The findings from section C, consisting of comparisons between existing care and potential care with the RADAR-CNS RMT system in different UPOC scenarios, are summarized below.

#### UPOC 1: Patient Sharing Data With Clinician

Respondents described that currently, information from patients is conveyed at face-to-face appointments or by phone but that RMT could permit transfer of data to a patient health information system before an appointment, permitting the automatic creation of a report. Clinician respondents suggested that this could be done over an encrypted channel to ensure data security.

Survey responses also indicated that the conventional means for aggregating data was via a manual patient diary; however, it was thought that the RADAR-CNS RMT system could summarize data automatically. The patient may have access and filter the data for relevance beforehand. In terms of clinical utility, RMT might replace or supplement conventional measures made in the clinic (eg, disability measures in MS), with the added advantage in epilepsy to overcome underreporting of seizures and thereby better inform medication adjustments.

In terms of timing, RMT permits data submission (by patients) and data analysis (by clinicians) at convenient times. However, concerning workload, respondents noted the potential for added burden to the clinician or, more generally, the health care provider organization, outside of appointment times.

#### UPOC 2-4: Relapse

Clinicians expected that the RADAR system would allow them to see if a relapse occurs; however, there was concern about both sensitivity and specificity, for example, that the relapse signal might be a false positive arising from another illness like influenza or that a true relapse may be missed (offering false reassurance). In terms of immediacy, respondents mentioned a delay in consultation without RMT either to a clinic appointment or a phone call from the clinician, whereas with the system, the emergency clinical service could receive an automatic alert (for depression relapse) or else prompt a clinician contact if an early sign of relapse had occurred (MS), employing a digital dashboard for the clinical team to manage this for all their patients.

It was considered that RMT could afford the opportunity to start an immediate intervention via a smartphone or else afford an earlier intervention than was possible without it. In terms of health care system benefits, there was a concern that data from RMT could overload or overburden the system; however, it could also benefit the health care system by reducing routine clinic visits for patients who are well and substitute routine visits by information provided remotely using RMT.

For a patient with a recent diagnosis having symptoms of relapse (UPOC 3), respondents described that the conventional approach was to see the patient in clinic, and this would be essentially unchanged with RMT, except for the potential for more continuous monitoring, resulting in a blended solution. As mentioned above, the main method for interaction with RMT would be via a clinician or team dashboard. For a very new depression diagnosis, RMT could be used to provide patients with access to information about their condition. For epilepsy, the system would detect closely spaced seizures or physiological markers indicating imminent relapse. For MS, it was also judged that it would take time to understand the symptoms in context from the RMT data, for example, variation due to the weather.

For a patient with a longer-term diagnosis having symptoms of a relapse (UPOC 4), the picture was similar to the above, but because a patient’s relapse pattern would more likely be known, the additional RMT data might prompt a change in the treatment plan. There was an additional general concern (not specific to RMT) that communication with the patient about a new depressive episode could increase the likelihood of depressive symptoms.

In general, longer-term data should enable more remote consultation, for example, by a phone call rather than a clinic visit, and a faster response.

#### UPOC 5 and 6: Medication Management

This section of the survey asked respondents to consider medication management before and after the imagined implementation of the RADAR-CNS RMT system, first in a new diagnosis of a CNS condition (UPOC 5) and second when a patient had lived with a condition for a long time (UPOC 6), with a view to examining differences in practice or perception. The results showed little difference between these 2 phases of conditions; thus, the results have been presented together.

The added benefit of RMT for newly diagnosed patients was considered to be low, as medication reviews would still need to happen face to face. Without RMT, people with epilepsy would write their own notes and wait for a change to a drug prescription, whereas with RMT, this could be done more efficiently via a mobile app. For depression, behavioral or social problems were considered difficult to detect remotely, although clinical information sent by the patient via RMT was envisaged as a way to trigger scheduling of an appointment. For MS, it was considered that the system might replace clinical scoring and magnetic resonance imaging, and fluctuations in disease activity might prompt a change in therapy. For a longer-term patient (UPOC 6), the results were mostly the same as those for UPOC 5, but one clinical respondent thought a change in treatment for a longer-term MS patient might be enacted via a phone call and the platform used to monitor the effect, rather than face to face (which they would recommend for a new patient).

### Sections D-F

Respondents were asked about the use of RMT data in clinical practice in general and were then asked about patient stratification and treatment response as 2 areas of interest. Areas of use of RMT data included improvement of clinical decision making and the enabling of more personalized care. RMT would be used to identify individual baselines or norms and individual patterns of behavior. It could also help discover population-level risk factors in MS and epilepsy, although it might not be as useful in the latter due to greater variation between individuals. For depression, RMT data could help educate the patient even if it were not possible to identify relapse signatures for individuals. It was considered, by a technical team respondent, that anomaly detection algorithms used in finance and industry could be used for RMT data to detect both individual- and population-level patterns. More research is needed, including the results of observational clinical studies, to determine whether RMT could be employed in therapeutic trials and to confirm whether it has a positive impact on individual disease management and patient outcomes.

Focusing on stratifying patients by risk or clustering in general, there were differences between specialties. In epilepsy, one example was dividing patients with more hazardous, frequent convulsive seizures from those with frequent focal seizures. For MS, RMT could allow clinicians to confirm a set of risk factors in the general MS population and determine how many of those risk factors are present for an individual patient. For depression, it was considered that RMT data could be used to test different treatments (on different groups) and potentially to stratify by genetic risk. It was considered that more research would be required to see how much data are needed to discover population risk factors and to determine if the population data are informative at the patient level for individual management.

### PAB

A total of 7 members of the PAB completed the survey as potential users of the system and provided a range of views on the benefits of RMT, mentioned personal areas of interest for using the technology, and raised some concerns. These 7 members included 2 members with experience living with depression, 2 members with epilepsy, and 3 members with MS.

Like clinicians, patients emphasized the importance of collaborative decision making about medication and highlighted across all 3 conditions the need to maintain face-to-face appointments even with the introduction of new technologies. However, with an RMT system in place, patients saw potential in having clinicians view their records between appointments, allowing side effects to be detected early on, and medication changes to be instigated earlier.

Patients also gave specific examples of signals that might be of interest, such as spelling mistakes and typing speed on mobile phones in MS. These points were not raised by health care professionals. There was also interest in considering potential negative effects arising from RMT use. For example, people with MS highlighted that they may not want to be informed by RMT that their condition was deteriorating, and people with depression mentioned that they prefer not to think about their condition between their 6-monthly clinical appointments.

Patients explained that they currently relied to a high degree on verbal communication with clinicians and their own memories of what had occurred between appointments. They acknowledged that these memories may not always be accurate, particularly in the case of epilepsy where some seizures may go undetected, and that the use of the system could provide more accurate data to base clinical decisions on. On the other hand, they also suggested that there may be a risk of increased anxiety due to tracking.

## Discussion

### Formative Development of the System via UPOC Methodology

**Figure 2:**
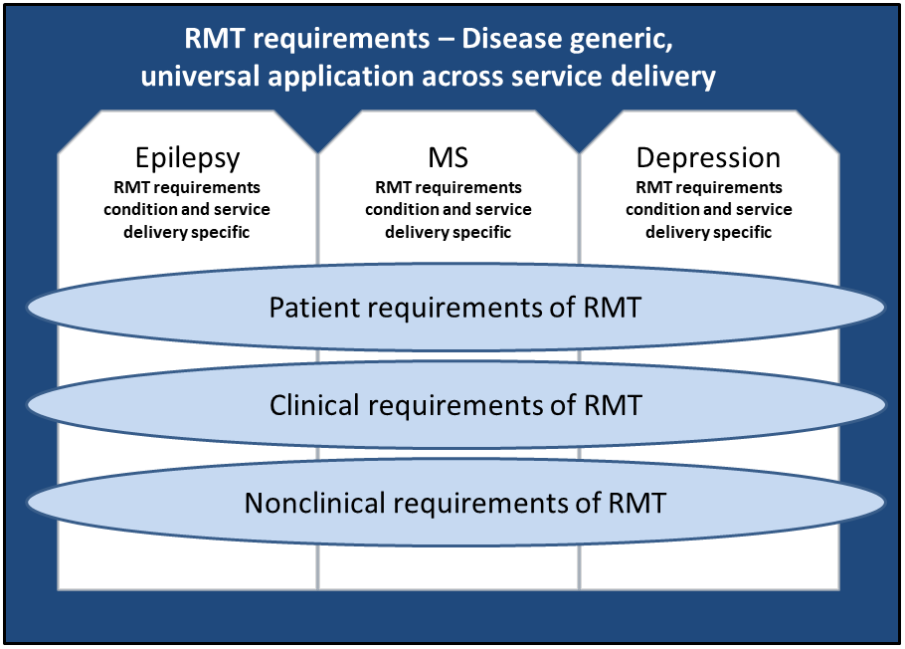
Diagram showing the coverage of the universal points of care methodology in the development of a novel remote measurement technology system. MS: multiple sclerosis; RMT: remote measurement technology.

From a methodological perspective, UPOC have been effective in eliciting condition-specific descriptions of current and potential processes from clinicians and patients. Figure 2 illustrates the coverage of the UPOC methodology across conditions and stakeholder requirements. Although the survey form provided was identical in all cases, participants were able to describe aspects of care within their relevant condition with details on their particular context, providing a rich set of data to support analysis of need, opportunity, and concern within and between conditions. For example, it was suggested that RMT should enable recognition of early warning signs of relapse, including identifying subclinical signals for MS, seizure precipitant signals for epilepsy, and behavior change in people living with depression. In terms of clinical utility, it was considered that RMT might overcome the problem of underreporting, which is especially problematic in epilepsy. It was also thought that RMT may allow the capture of secondary symptoms that are not generally collected in MS, such as mood.

Several novel and unanticipated uses of RMT were suggested by respondents. For depression, it was considered that RMT could help extend investigations into psychotherapies and digital mental health interventions. For epilepsy, active remote measurement technology with questionnaires could also be used to detect emerging side effects of treatment, although care would need to be taken to understand the responses correctly before acting. It was considered that RMT could enable behavior change for sleep regulation and activity and that it could be helpful in detecting minor relapses for people with cognitive impairments who may not mention them in the clinic and thus help prompt or guide escalation of care. RMT was also considered to offer a way to include family and other carers in a care network, which could assist with goal setting, although with care to avoid the risk of overreliance on information from carers, which may not be reliable. Although the current iteration of the system was not designed to feed data or analyses from the system to the patient, this is an aspect of usage that could be added to future iterations to facilitate the operations described here.

The novel adaptation of a medical device usability model [7] with its 4 embedded qualifiers (*enabler*, *enhancer*, *facilitator,* or *catalyst*) helps developers understand more precisely how patients perceive value in the system being designed. In this study, the method was used to structure the results of the end user inquiry. The method could also be used in summative evaluation to test whether the promise of improvement is realized at key stages of an iterative design process.

### Comparison With Related Work

This study included patients and research clinicians to contribute a multistakeholder perspective considering where and how RMT might improve care in 3 conditions, which has been described as lacking in a review of the literature in this area [9]. In particular, this study has added to the existing work by demonstrating how clinicians and patients view the promise of RMT as a way of altering clinician-patient interactions. Previously, the clinician has been seen as the main decision maker and expert provider of information and knowledge in the clinician-patient relationship [2,3]. Both parties envisaged ways that patients could act as custodians of their own data (eg, by being able to access and manage it) but they also emphasized that enabling the remote transmission of data should not result in the removal of all face-to-face interactions, a finding that is supported by other work in this area [30].

The consortium has also explored wider patient views of RMT in the 3 conditions [5,31,32]. Although these studies focused on the barriers and facilitators of the use of RMT, this study involved members of the PAB who had significant knowledge of the project, enabling the collection of their ideas on particular features in the data collected from the RMT that could be used for monitoring the deterioration of their own condition. For example, PAB members made the observation that in MS, spelling mistakes and typing speed on mobile phones may be of use in detecting changes in their cognitive state. There was also interest in detecting early side effects of medications, permitting the earlier change of medication where this was problematic. Thus, patients themselves were empowered in this study to speak from their own insight and suggest how RMT could be serviceable to them both in detecting changes in their condition and managing potential for harm.

In addition to this study, we have conducted further work with a wider community of health care professionals outside the RADAR-CNS consortium in the form of an interview study to gauge their views on the potential of RMT [33]. Whereas the interviews in the study by Andrews et al [33] aimed to elicit the types of data, specific time points, and job roles where RMT data would be used, the use of UPOC in this study asked participants to be more creative and imagine how particular processes could be differently managed with the use of RMT. Thus, responses in this study permitted capture of imagined scenarios of use for RMT at specific points of care, for example, how a change in treatment for MS might be enacted via a phone call and the platform used to monitor the effect. The results here are therefore more indicative of how clinical pathways might change to accommodate the use of these technologies than in the results of our previously published work, and the results of this study incorporate both clinician and patient perspectives.

### Limitations and Future Work

The results of this study are limited due to the population sample, albeit by design, with its primary focus on health care professionals and patient representatives from a research consortium where one could presume most to be enthusiasts of the technology being developed. In this study, the use of UPOC has guided clinicians and patients to portray their own views of how RMT should augment care. Within these portrayals, participants were able to articulate where further inquiry was necessary to achieve such improvements. Participants were of the opinion that more research is needed to assess the sensitivity and specificity of relapse prediction and that future prospective trials should take place to assess the impact on clinical care and patient outcomes. It is too early to say whether RMT could be employed successfully in therapeutic trials or whether it can really support individual disease management and stratification of patients based on risk factors or treatment response.

Despite the benefits of our approach, responses in some areas were not detailed enough to design a system based solely on the outputs of this study. For example, it is not yet clear exactly how the alerts and follow-up appointments that are triggered by RMT will be managed in routine care, for example, would they be directed first to patients, carers, or clinical services? Would they generate automated (eg, programed behavioral advice) or human responses? The survey did not reveal the details of information that health care professionals require from the system, for example, the required sensitivity and specificity thresholds for alerts. However, it is clear that the UPOC method was able to reveal the need to consider such details.

Beyond the scope of this study, the UPOC approach provides learning for the design community, specifically those working in the health and medical domains and in the development of complex Information and Communication Technology platforms. The approach taken to identify and design a requirements capture exercise around UPOC has shown that formative data capture is possible and will provide important insights into how multifaceted platforms can meet the needs of different stakeholders from different clinical specialties, for whom there are different end goals.

### Conclusions

The UPOC method employed in this study provided a targeted and structured means by which to inquire about use of RMT in real-world practice and bring clinical and patient opinion into its design process. In addition, by adapting the model by Sharples et al [7] to label solutions as enablers, enhancers, facilitators, or catalysts, we were able to offer signposting to technologists about how different aspects of the digital platform could provide value in a number of different use case scenarios.

More generally, the UPOC approach provides a means to explore the requirements of a complex technology system that not only needs to meet the broad needs of traditional user groups, such as health care professionals and patients, but also has to accommodate the diverse experiences of those groups of stakeholders from the range of clinical specialties within them. A greater understanding of these needs will inform a meaningful formative and summative evaluation of system design.

## Appendix

Multimedia Appendix 1 Survey instrument used the present study.

Multimedia Appendix 2 Themes derived from survey section B on the promise of remote measurement technology.

## Abbreviations

CNS: central nervous system
EFPIA: European Federation of Pharmaceutical Industries and Associations
HITs: health information technologies
IMI: Innovative Medicines Initiative
MS: multiple sclerosis
NHS: National Health Service
NIHR: National Institute for Health Research
PAB: patient advisory board
RADAR-CNS: Remote Assessment of Disease and Relapse-Central Nervous System
RMT: remote measurement technology
UPOC: universal points of care
