# Supplementary material for: Informing the Development of a Digital Health Platform Through Universal Points of Care: Qualitative Survey Study
Source: JMIR Form Res. 2020 Nov 26;4(11):e22756. doi: 10.2196/22756 (PMC7728533; doi:10.2196/22756)
Supplement: Multimedia Appendix 1 [file formative_v4i11e22756_app1.pdf]

RADAR-CNS Internal Consortium Survey

Investigating how RADAR-CNS might be used in clinical practice

WP09 are looking to elicit 'use scenarios' from clinical WPs and PAB members. This will help inform enquiries that are external to the consortium and contribute important information about how RADAR-CNS may be integrated into clinical practice in the long term.

We look forward to receiving responses from patients & service users, clinicians, researchers and academics. All contributions will be recorded anonymously within a separate document.

If you have any queries then please contact one of the team from WP09 or Sara from WP02.

Alex Lang – Alexandra.Lang@nottingham.ac.uk

Mike Craven – Michael.Craven@nottingham.ac.uk

Sara Simblett - sara.simblett@kcl.ac.uk

Section A: Participant Information

Participant Name: ____

Participant WP: ____

PAB member: Yes / No (please select) ____________________________________

Institution/Company: ____ (if relevant)

Participant job title: ____ (if relevant)

Medical Specialism/Condition: ____

Section B: What do you think RMT will offer care provision in MS/Epilepsy/Depression?

Please answer specifically to the medical domain/condition you listed in Section A.

**1. In your medical domain/condition, what are the questions that you think RMT will answer?**
**2. Within the scope of the RADAR-CNS project what are the essential questions that need to be answered by the end of the observation trials?**
**3. Within the scope of the RADAR-CNS project what are the desirable questions that you would like to see answered by the end of the observation trials?**

Section C: Current Clinical Practice vs Clinical Practice with RADAR-CNS

This question explores how you think the RADAR-CNS system might work as an integrated part of clinical practice.

We have provided some generic situations in clinical practice that you will be familiar with, either as a clinician or as a service user.

We would like to understand what happens in current practice and also what you think might happen in this situation if the RADAR-CNS system was in use.

In your answers, please think about the following and provide as much detail as possible.

- Where does the interaction take place? Face to face? Virtually?
- Are any communications in 'real time' or is there a delay?
- What are the positives about this way of interacting?
- What are the negatives about this way of interacting?

**Task 1**<br><br>**Patient sharing data with clinician**<br><br>A patient has been monitoring how they feel (activity, mood, sleep etc) for two weeks. They have some concerns and would like to share the data with their clinician. How might they share their data?	How does this happen in current practice **without** RADAR-CNS?	How might this happen **with RADAR-CNS** in use in clinical practice?

**Task 2**<br><br>**Relapse Detection**<br><br>A patient is not feeling well and might be on the path to a relapse. How do you	How does this happen in current practice **without** RADAR-CNS?	How might this happen **with RADAR-CNS** in use in clinical practice?

(clinician or patient) try to understand if a relapse is imminent? How do you want information to come to you?		

**Task 3**<br><br>**Relapse Detection, communication with *patient who has been recently diagnosed***<br><br>The patient might be headed for a relapse. The clinicians and patients have not been working together for very long and the patient is still getting used to their diagnosis. How would you like to interact and communicate in this situation?	How does this happen in current practice **without RADAR-CNS**?	How might this happen **with RADAR-CNS** in use in clinical practice?

**Task 4**<br><br>**Relapse Detection, communication with *patient who has had their diagnosis for a long time***<br><br>The patient might be headed for a relapse. The clinicians and patient know each other well and the patient has been living with the condition for a long time. How would you like to interact and communicate in this situation?	How does this happen in current practice **without RADAR-CNS**?	How might this happen **with RADAR-CNS** in use in clinical practice?

Task 5<br><br>**Medication selection/dosing *in a patient who has recently been diagnosed***<br><br>The patients' medication needs modifying. What is the best way to inform them of this decision and provide them with the information?	How does this happen in current practice **without RADAR-CNS**?	How might this happen **with RADAR-CNS** in use in clinical practice?

Task 6<br><br>**Medication selection/dosing *in a patient who has had their diagnosis for a long time***<br><br>The patients' medication needs modifying. What is the best way to inform them of this decision and provide them with the information?<br><br>This could include evaluating medication response, planned medication withdrawal/ discontinuation.	How does this happen in current practice **without RADAR-CNS**?	How might this happen **with RADAR-CNS** in use in clinical practice?

Section D: Clinical Use Scenarios for RADAR-CNS

Can you think of any other ways in which the RADAR-CNS system might impact clinical practice?

Are there any other situations where use of the technology might change your decisions, behaviours or experiences or the way in which you interact with other people (clinicians, patients, other healthcare professionals).

We are interested in knowing about these situations (also called use scenarios). We would like to understand your perspectives, expectations and needs of the RADAR-CNS system in these situations.

Please use the two scenario boxes below to try and think about some of these situations and describe them in as much detail as you can. (If you want to provide more than two then please feel free to email Alex in WP09 with your additional ideas).

Scenario 1
**Brief Description –**
**Context and Environment of use -**
**Users/Stakeholders -**
**Expected or likely impact on clinical practice (any changes to current practice?) –**
**Concerns or potential risks associated with this scenario –**

Scenario 2
**Brief Description –**
**Context and Environment of use -**
**Users/Stakeholders -**
**Expected or likely impact on clinical practice (any changes to current practice?) –**
**Concerns or potential risks associated with this scenario –**

Section E: What is your perception about how the data will be used in practice?

1. What are your thoughts about the use of RADAR-CNS data for individual patient management vs population level analysis?<br>In your answer to this, please consider and comment on,<br>- RADAR-CNS data for understanding individual changes<br>- RADAR-CNS data for benchmarking/comparing to a norm
**2. What are your thoughts about the use of RADAR-CNS data for understanding and stratifying patient risk?**

3. What are your thoughts about the use of RADAR-CNS data for stratifying responders and non-responders to treatment?

Section F: Do you have any other thoughts regarding any of the topics raised in the survey or additional related content?

Thank you for taking the time to fill in this questionnaire.
