# Supplementary material for: Informing the Development of a Digital Health Platform Through Universal Points of Care: Qualitative Survey Study
Source: JMIR Form Res. 2020 Nov 26;4(11):e22756. doi: 10.2196/22756 (PMC7728533; doi:10.2196/22756)
Supplement: Multimedia Appendix 2 [file formative_v4i11e22756_app2.docx]

**Themes derived from survey section B on the promise of remote measurement technology.**

| **Final theme** | **Promise** |
| --- | --- |
| Relapse prediction | Enable: recognition/warning of signs of relapse; identification of sub-clinical [MS]/seizure precipitant signals [E]; creation of prediction models; use of sensor data [D]  Facilitate: personalising of relapse signatures [D] |
| Clinical verification | Enable: quality of life measurement; potential for verifying clinical functions with respect to gold standards; measurement of deterioration of motor system in progressive MS [MS]; type of epileptic seizure [E]; when did seizure occur [E]; recognition of related phenomena associated with relapse e.g. bradycardia, convulsions that might be prospectively predictable and/or modifiable [E]; putting the seizure occurence into context of prior events [E].  Enhance: more precise measures of function e.g. disability; (more) detailed characterization of MS progression [MS]; insight into complex function in daily life not covered by routine clinical scales. |
| Monitoring potential | Enable: covering of time between treatment sessions; daily assessments; safety related identification; convulsion when alone [E]; seizure in a dangerous environment [E].  Enhance: more ecological assessment of emotional and physical state of a patient; More valid assessment of seizure frequency and timing [E].  Catalyse: identifying change of state more quickly to allow earlier intervention |
| Patient behaviour | Enable: physiological modification and alteration in daily life; discover what patient is doing in-between treatment sessions  Enhance: increase the active engagement of patients; increase self-efficacy and/or self-awareness of patients; develop personal sensing algorithms that use sensor data to estimate behaviours  Facilitate: Compliance/adherence to RMT |
| User experience | Enable: gain insight in the feasibility and acceptability/willingness to use of RMT; RMT feasible for old[er] patients? Can patients with physical disabilities, e.g. reduced mobility and impaired visual perception skills, complete questionnaires and execute cognitive tasks by smartphone? |
| Changes to treatment approach | Enable: physiological modification and alteration in daily life; find the predictor factors of the relapse and improve the therapeutic adherence of the patients. |
| Clinical decision-making | Enable: detailed characterization of MS progression to be used in clinical decision making [MS]; existing or evolving problems with mood etc. that could be predictable and/or modifiable? [E] |
| Clinical trials design | Enable: detailed characterization of MS progression to be used in designing clinical trials [MS] |
| Device issues | Does [device] track the steps and activity correctly? Selection of potential devices to be used in these studies. |

**Supplementary Table 1**. Themes derived from survey section B on the promise of RMT. Theme content is labelled with condition as follows: [MS] – multiple sclerosis, [E] – epilepsy, [D] – depression.
